# Aortotracheal fistula after slide tracheoplasty in a patient with dextrocardia, left pulmonary artery sling and tracheal stenosis: a case report

**DOI:** 10.1186/s13019-021-01438-6

**Published:** 2021-03-31

**Authors:** Yu-san Chien, Yen-Chun Chao, Kuo-Sheng Lee, Kang-Hong Hsu

**Affiliations:** 1 Department of Critical Care Mackay Memorial Hospital, CVICU-B, 7F, No. 92, Sec. 2, Zhongshan N. Rd, Taipei City, Taiwan; 2 Department of Pediatric Cardiology Mackay Children’s Hospital, PICU, 11F, No. 92, Sec. 2, Zhongshan N. Rd, Taipei City, Taiwan; 3 Department of Otorhinolaryngology and Head & Neck Surgery Mackay Memorial Hospital, No. 92, Sec. 2, Zhongshan N. Rd, Taipei City, Taiwan; 4 Department of Cardiovascular Surgery Mackay Memorial Hospital, CVICU-B, 7F, No. 92, Sec. 2, Zhongshan N. Rd, Taipei City, Taiwan

**Keywords:** Aortotracheal fistula, Slide tracheoplasty, Dextrocardia, Case report

## Abstract

**Background:**

Aortotracheal fistula (ATF) is an uncommon and fatal complication of tracheal or aortic surgery, especially among pediatric patients.

**Case presentation:**

We reported a case in a 1-year-old boy with dextrocardia, left pulmonary artery sling and long segment tracheal stenosis. He received slide tracheoplasty at 9 months of age and had post-operative refractory granulation at distal trachea status post repeated balloon dilatation and laser vaporization. Episodes of hemoptysis occurred on post-operative day 81. Bronchoscopy revealed a pulsating pseudoaneurysm at lower trachea which ruptured during the procedure

Urgent surgical repair under cardiopulmonary bypass with deep hypothermic circulatory arrest was done. No recurrent bleeding or significant neurologic deficits noticed at a 4-month follow-up.

**Conclusion:**

Congenital anomaly that changes the spatial relationship between trachea and aorta could have contributed to formation of ATF. This warrant future attention when managing tracheal granulation with this not uncommon anatomy.

**Supplementary Information:**

The online version contains supplementary material available at 10.1186/s13019-021-01438-6.

## Background

Aortotracheal fistula (ATF) is a rare and potentially fatal complication of aortic or tracheal surgery [1–3]. An early review of 62 adult patients found that 86% of the fistulas were between descending aortas and left bronchopulmonary trees [4]. An ATF involving aortic arch is uncommon and could considerably increase the difficulty of treatment. Here we presented an unusual case of ATF in a 1-year-old boy with dextrocardia, left pulmonary artery sling and tracheal stenosis status post re-implantation of left pulmonary artery and slide tracheoplasty.

## Case presentation

A 1-year-old boy presented with intermittent hemoptysis during hospitalization in our pediatric intensive care unit. He had history of left pulmonary artery sling, long segment funnel-shaped tracheal stenosis (O-ring with the smallest inner diameter of 2.5 mm), and dextrocardia. Due to frequent lower airway infections and progressive respiratory distress, re-implantation of left pulmonary artery and slide tracheoplasty were done at 9-months-old. His trachea was transected 2.5 cm above carina and was shortened by approximately 1 cm after surgery. His post-operative course was complicated by tracheobronchomalacia and restenosis of distal trachea caused by granulation tissue at anastomotic sites. Balloon dilatation, Holmium laser vaporization and granulation tissue removal were performed five times but with limited effects. The patient experienced four unsuccessful extubation within 2 months.

**Fig. 1:**
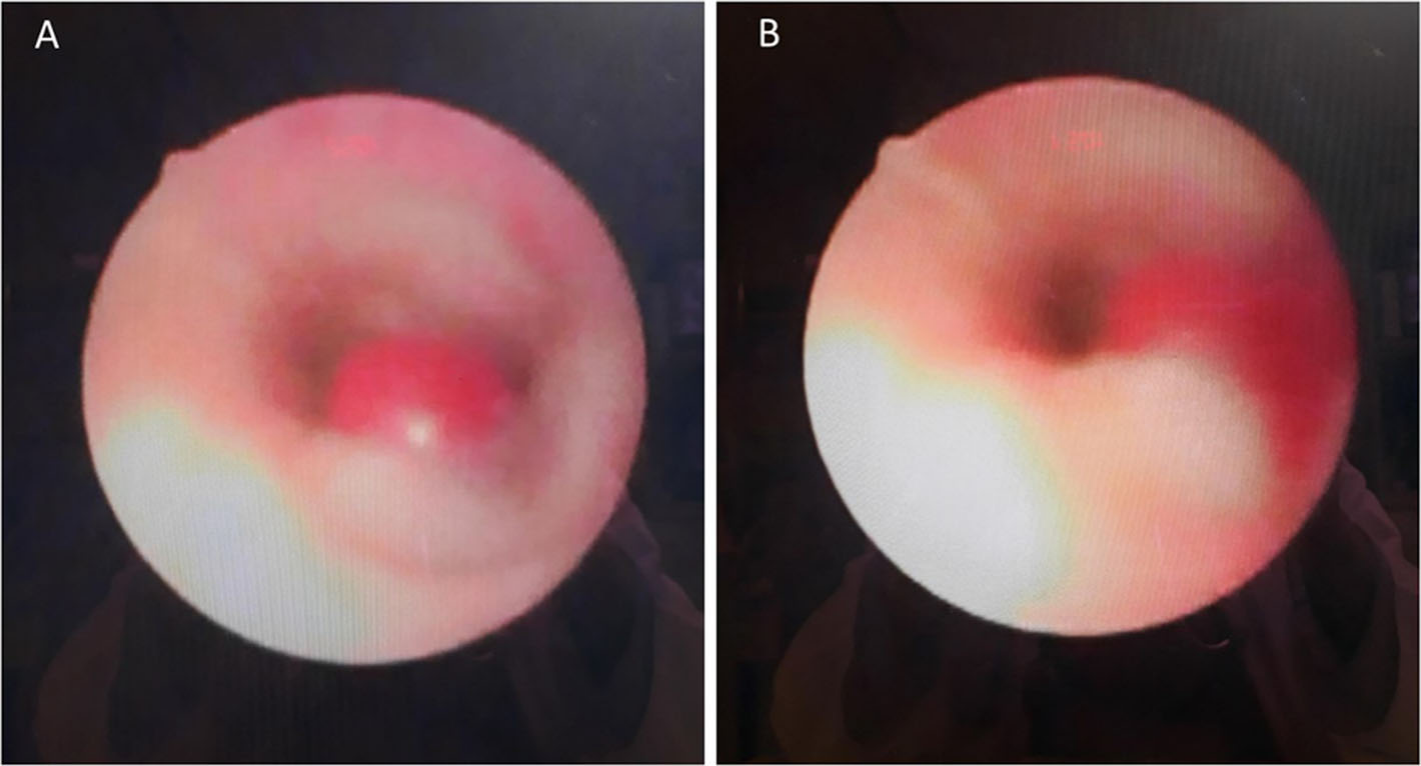
Bronchoscopy showed a pulsating pseudoaneurysm at anterior wall of lower trachea (**a**) with witnessed rupture during the procedure (**b**)

**Fig. 2:**
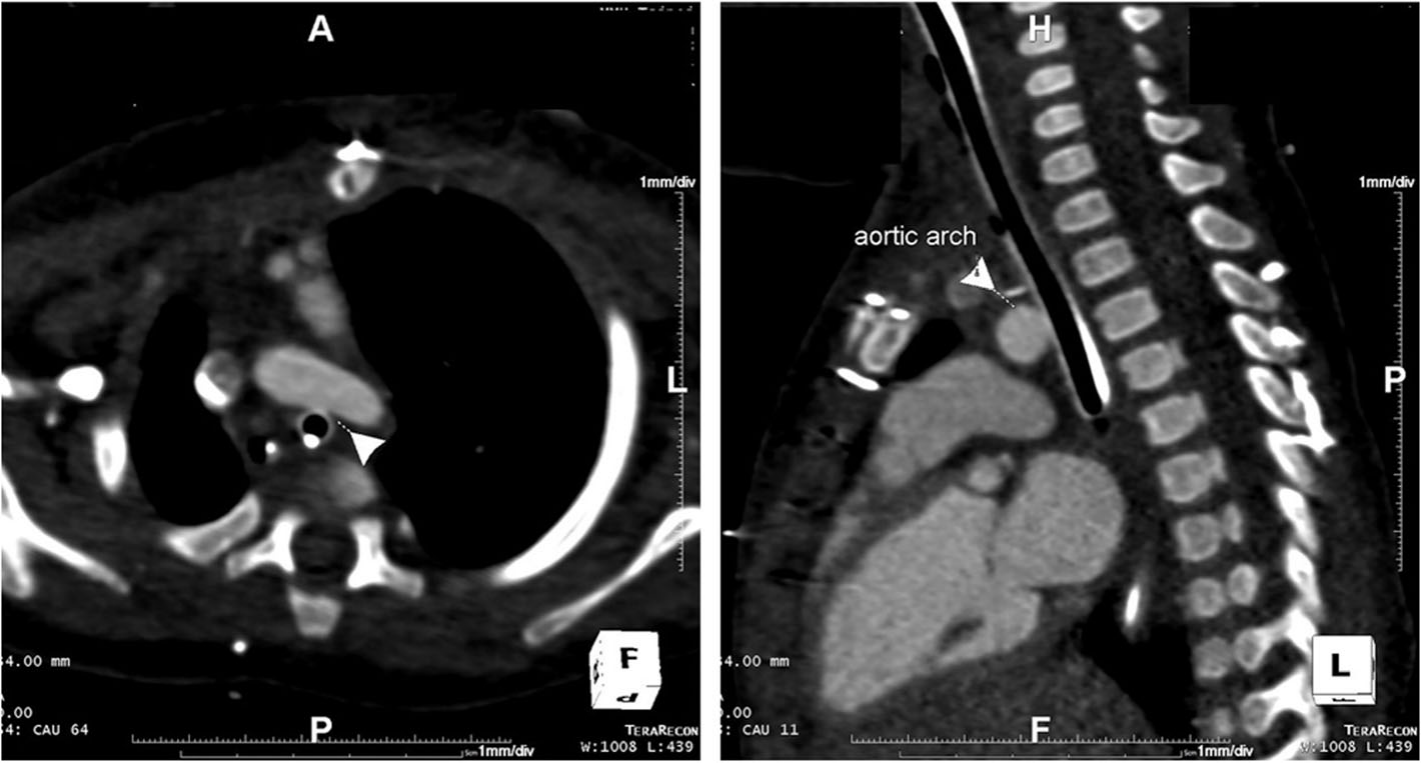
Contrast-enhanced computed tomography angiography (CTA) showed poor differentiation of the borders between posterior wall of distal aortic arch and anterior wall of lower trachea, but with no contrast extravasation into airway (white arrow indicates aortic arch)


On post-operative day 81, the child presented with hemosputum, airway obstruction and hypercapnia. Diluted epinephrine was repeatedly administered via endotracheal tube and the bleeding resolved. Laboratory examinations revealed normocytic anemia and a normal coagulation profile. Minimal infiltrates over left lower lung field was seen on chest radiograph. Rigid bronchoscopy performed 8 h later did not find bleeding foci despite some blood clots visible in distal trachea and both main bronchus orifices. Technetium-99 m-labeled red blood cell scan on the same day did not show abnormal radiotracer accumulation in the chest. Another episode of hemoptysis occurred 2 days later. Under guidance of fiberoptic bronchoscopy, endotracheal tube was withdrawn, and a protruding pulsating lesion appeared at anterior wall of lower trachea. Witnessed rupture of the suspected pseudoaneurysm with concomitant massive bleeding occurred during the procedure (Fig. 1, Video). Endotracheal tube was placed back immediately for direct compression and bleeding temporarily stopped. Contrast-enhanced computed tomography angiography (CTA) showed poor differentiation of the borders between posterior wall of distal aortic arch and anterior wall of trachea 1.5 cm above carina but no contrast extravasation into airway (Fig. 2). Combined the findings of bronchoscopy and CTA, aortotracheal fistula was impressed and the patient was taken to operation theater for urgent repair.

**Additional file 1:** Video.

After median sternotomy, the child was put on cardiopulmonary bypass (CPB) by cannulation to ascending aorta and right atrium. The patient was cooled down to 18 °C for possible circulatory arrest during aortic arch repair. His proximal aorta was cross clamped and antegrade cardioplegia was given. Deep hypothermic circulatory arrest (DHCA) was initiated with selective antegrade cerebral perfusion (SACP) by advancing the arterial cannula to innominate artery with a flow at 50 ml/min/kg and a right radial artery pressure around 50 mmHg. Near-infrared spectroscopy was used for monitoring bilateral cerebral perfusion. Because of severe adhesion between aortic arch and reconstructed trachea, intra-luminal approach of the fistula with an incision along the aortic arch curvature was tried first. A 2 mm defect was identified on the posterior wall of aortic arch with a thin membranous structure left between arch and trachea. Direct closure of fistula ostium with 6–0 running proline sutures was done. During rewarming, intra-operative bronchoscopy showed much fresh blood in the patient’s airway. He was then cooled down to 18 °C with DHCA with SACP again. After meticulous dissection of aortic arch from trachea and surrounding firm adhesions, a generous autologous pericardial patch was used to cover the posterior wall of aortic arch and to seal the defect. During rewarming, tracheal defect was repaired using another autologous pericardium. No more bleeding was identified by the bronchoscopy. Post-operative course was uneventful. No recurrent bleeding happened, and no significant neurologic deficits noticed at a 4-month follow-up. The patient passed away on 2 months later due to airway obstruction and ventilation failure caused by recurrent refractory tracheal granulation.

## Discussion and conclusions

Information regarding the epidemiology, pathogenesis, and management of aorto-tracheal fistula is scarce, probably because this condition is so fatal that most patients succumb before any treatment can be initiated. An adult series in 2014 showed an incidence of aorto-bronchial fistula of 0.56% after thoracic endovascular aortic repair [5]. Another report in 2003 found 1 out of 51 children who underwent major reconstructive tracheal surgery had ATF [1]. But the actual incidence of ATF remains unknown, especially among pediatric patients.

**Fig. 3:**
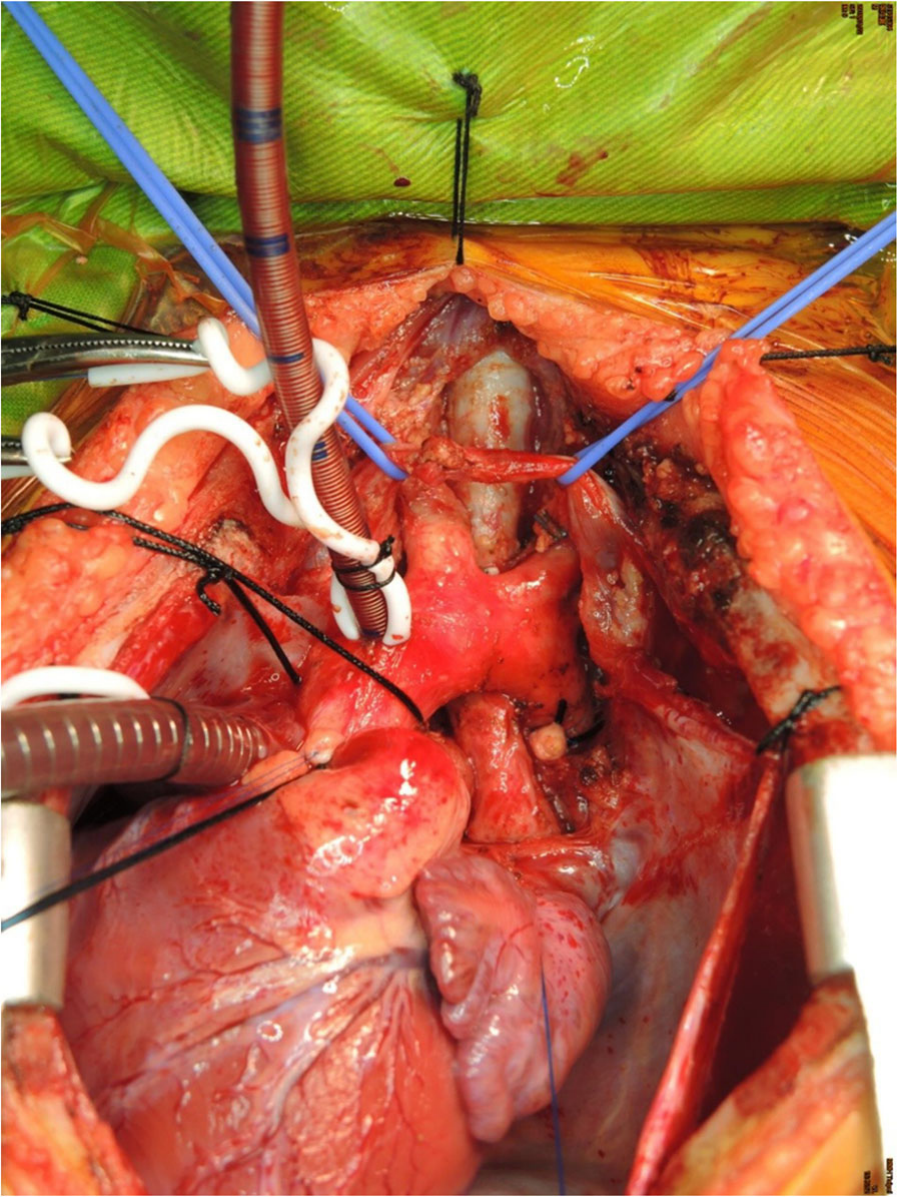
Dextrocardia with left aortic arch sitting across his lower trachea

In terms of pathophysiology, catastrophic bleeding from fistulas between great vessel and major airway often involves pressure necrosis or chronic infection / inflammation of tracheal mucosa [6, 7]. Congenital anomalies that change the anatomic relationship between trachea and aorta had been reported to be associated with ATF, such as double aortic arch, and left pulmonary artery sling [1, 8, 9]. Our patient has dextrocardia, which resulted in compression of his lower trachea by distal arch (Fig. 3) and received repeated laser ablation and balloon dilatation for tracheal stenosis at similar level. Both could have contributed to the near fatal event and warrant future attention when managing refractory tracheal granulation with this not uncommon anatomy.

Sentinel bleeding has been reported as a dangerous sign in up to 35% of tracheoinnominate fistula [10] and had happened in our patient. Despite the critical nature of aortotracheal fistula, bronchoscopy or tagged red blood cell scan performed hours after the episode of hemorrhage could not identify the etiology. CTA could delineate the anatomic relationship between airway and vascular structures, but it did not clearly demonstrate the fistula either. Real time bronchoscopy was risky but seemed to be more helpful in making diagnosis. A contingency plan must be in place in case massive bleeding occurs.

Unlike fistulas involving descending aorta or innominate artery that could be successfully treated through direct ligation or endovascular repair [11, 12], full support with cardiopulmonary bypass and deep hypothermic circulatory arrest were required for surgical intervention in our patient because the suspected defect located at transverse aortic arch. From our experience, direct closure of the vascular defect could lead to greater tension of the sutures and therefore unable to achieve a complete hemostasis. One patch method with only a thin layer between great artery and the airway was not used considering a possible higher risk of pseudoaneurysm formation and sepsis caused by respiratory tract infection. Two patch method is recommended. During the procedure, arterial cannula was advanced to innominate artery for antegrade cerebral perfusion and his neurologic function was well preserved after the operation. This strategy could be adapted by other physicians when facing a similar scenario.

In conclusion, aortotracheal fistula may occur after repeated tracheal surgeries, especially in patients with congenital anomalies that change the anatomic relationship between major airway and great vessels.

## Data Availability

Not applicable.

## Abbreviations

ATF: Aortotracheal fistula
CTA: Contrast-enhanced computed tomography angiography
CPB: Cardiopulmonary bypass
DHCA: Deep hypothermic circulatory arrest
SACP: Selective antegrade cerebral perfusion
